# Reproduction-Associated Hormones and Adult Hippocampal Neurogenesis

**DOI:** 10.1155/2021/3651735

**Published:** 2021-09-10

**Authors:** Lily Wan, Rou-Jie Huang, Zhao-Hui Luo, Jiao-e Gong, Aihua Pan, Jim Manavis, Xiao-Xin Yan, Bo Xiao

**Affiliations:** ^1^Department of Neurology, Xiangya Hospital, Central South University, Changsha, Hunan 410008, China; ^2^Medical Doctor Program, Xiangya School of Medicine, Central South University, Changsha, China; ^3^Department of Neurology, Hunan Children's Hospital, Changsha 410007, China; ^4^Department of Anatomy and Neurobiology, Central South University Xiangya School of Medicine, Changsha, Hunan 410013, China; ^5^Faculty of Health and Medical Sciences, The University of Adelaide, Adelaide, SA, Australia 5000

## Abstract

The levels of reproduction-associated hormones in females, such as estrogen, progesterone, prolactin, and oxytocin, change dramatically during pregnancy and postpartum. Reproduction-associated hormones can affect adult hippocampal neurogenesis (AHN), thereby regulating mothers' behavior after delivery. In this review, we first briefly introduce the overall functional significance of AHN and the methods commonly used to explore this front. Then, we attempt to reconcile the changes of reproduction-associated hormones during pregnancy. We further update the findings on how reproduction-related hormones influence adult hippocampal neurogenesis. This review is aimed at emphasizing a potential role of AHN in reproduction-related brain plasticity and its neurobiological relevance to motherhood behavior.

## 1. Introduction

**Figure 1:**
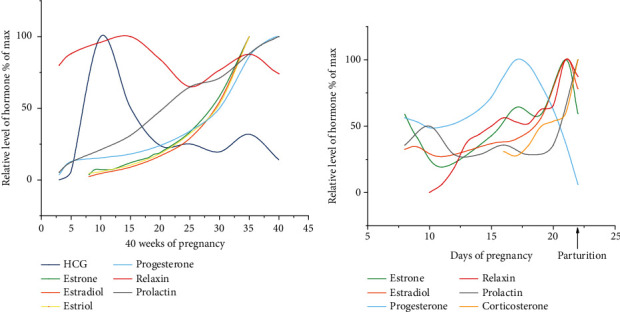
Schematic illustration of the changes in the levels of serum hCG [3], estrone [4], estradiol [4], estriol [4], progesterone [5], relaxin [6], and prolactin (PRL) [5, 7] during gestational weeks 6-40 in humans according to literature as indicated (the numbers in the *x*-axis indicate the gestational week). Blood samples were obtained through venipuncture of pregnant human females, and the diagnosis of pregnancy was confirmed by urine hCG determination and/or ultrasonography. HCG was determined via the double-antibody beta radioimmunoassay. Estrogens (estrone, estradiol, and estriol) were determined with an antibody against estrone-17-oxime coupled with bovine serum albumin. Progesterone was measured via the Bayer ADVIA Centaur assay. Relaxin was measured via radioimmunoassay with iodine 125-labeled polytyrosyl-relaxin and rabbit anti-porcine relaxin serum R6. PRL was determined via radioimmunoassay with homologous double antibodies. (b) Summary of the changes in the levels of estrone [8], estradiol [8], progesterone [9], relaxin [10], PRL [11], and corticosterone [12] in rat from gestational days 8-22 according to existing reports as indicated (the *x*-axis indicates the gestational days). The samples were derived from the peripheral blood of primiparous Sprague-Dawley rats (estrone, estradiol, progesterone, relaxin, and PRL) or the arterial blood of Wistar rats (corticosterone). Estrone and estradiol were determined via radioimmunoassay with estradiol-17*β* antiserum. Progesterone was quantified by protein-binding displacement. Relaxin was measured via the interpubic ligament bioassay. PRL was determined via radioimmunoassay. Corticosterone was measured using the fluorometric method.

During pregnancy, the levels of reproduction-related hormones, including human chorionic gonadotropin (hCG), estrogen, progesterone, relaxin, prolactin (PRL), and oxytocin (OT), exhibit sharp variations [1–12] (Figure 1). Striking changes in cognition and behavior also occur during pregnancy and postpartum, involving multiple neuropsychological aspects such as learning, memory, mood, and childcare. Studies in animal models such as rats, mice, and sheep have shown that hormonal changes during pregnancy and postpartum may significantly modulate the process of neuroplasticity, including adult neurogenesis, in many brain regions such as the hypothalamus and hippocampal formation [13–17].

Adult neurogenesis refers to the proliferation and differentiation of neural stem cells or neuron precursor cells into new neurons in the adult brain [18, 19]. So far, adult neurogenesis is considered to occur principally in the subventricular zone (SVZ) in the wall of the lateral ventricle and subgranular zone (SGZ) in the dentate gyrus of the hippocampal formation [20, 21], which has been known to play an essential role in olfactory and spatial learning and memory. Adult hippocampal neurogenesis (AHN) appears also important for other cognitive functions, mood regulation, and behavioral processes in normal conditions. Importantly, impairment of AHN is related to the pathogenesis of many psychiatric disorders and neurological diseases affecting different age groups [22–24].

The role of adult neurogenesis in shaping neuroplasticity thereby modulating reproduction or motherhood-related cognition and motherhood behavior is a topic of broad interest involving both the basic and clinical neuroscience fields. However, researches in the front remain fairly limited, with data from a number of animal species available. This review is aimed at updating the current understanding regarding the potential effects of reproductive hormones on adult hippocampal neurogenesis. We first briefly introduce the general functional significance of AHN and the methods commonly used to explore its role in vivo. Next, we attempt to reconcile the changes of reproduction-associated hormones during pregnancy based on animal and human studies. We further update the findings on how various reproduction-related hormones may influence AHN. Overall, this review attempts to emphasize a potential neurobiological role of AHN in reproduction-related brain plasticity that may modulate motherhood behavior.

## 2. Functional Studies on Adult Hippocampal Neurogenesis

### 2.1. Functional Significance of Adult Hippocampal Neurogenesis

AHN is considered to underlie numerous hippocampus-dependent fuctions, including learning and memory, spatial navigation, new memory formation, and old memory forgetting functions [25, 26]. The newly generated granule cells may contribute to significant structural and functional plasticity in the trisynaptic hippocampal circuits in response to internal and external stimuli thereby also fulfilling new functional demands [27].

AHN can be alteredin the context of stress, physical training, changes in the levels of multiple hormones, sleep deprivation, and even some diseases, excitement has been fueled regarding whether and how the neonatal neurons integrate into the relevant neural circuits and influence action and whether neurogenesis improves hippocampus-dependent memory and learning. Most studies to date have demonstrated that adult neurogenesis enhances hippocampal-dependent functions [26, 28]. For instance, several approaches have been adopted to assess alterations in spatial learning and memory, including low-dose irradiation, antimitotic treatment technology, and genetic modification to ablate adult newborn neurons. Mice submitted to these procedures exhibited pronounced impairment of certain cognitive functions after the adult newborn neurons were eliminated when the ablation was performed after memory formation [29, 30]. Zhang et al. treated mice in the postpartum estrogen withdrawal (EW) group with anti–N-methyl-D-aspartate receptor stimulants and the resting mice with inhibitors; the former exhibited a significant increase in BrdU-immunolabeled-positive cells coupled with an increased rate of depression and anxiety, whereas the latter did not [31]. The excitability and synaptic plasticity of the CA1 and CA3 regions of the hippocampus were enhanced when the estrogen level reached its maximum (proestrus), at which point the proliferation of hippocampal dentate gyrus cells increased [32]. Berdugo-Vega et al. reported that increasing neurogenesis refines hippocampal activity, thereby rejuvenating navigational learning strategies and contextual memory throughout life [29]. Furthermore, senescence is an important negative biological factor in hippocampal adult neurogenesis. Studies have shown that aging-associated loss of cognition can be reversed by increasing adult hippocampal newborn neurons [33]. In addition to aging, growing evidence has indicated that aging-related neurological diseases such as Alzheimer's and Parkinson's disease may impair adult hippocampal neurogenesis. Radad et al. demonstrated that adult neurogenesis has great therapeutic potential for the treatment of Parkinson's disease and Alzheimer's disease [34]. Additionally, depression and anxiety are also associated with adult hippocampal neurogenesis. Research has indicated that both hippocampal volume and adult hippocampal neural progenitor cells decreased in patients with depression, and antidepressant treatment increased the volume of the adult hippocampal dentate gyrus and the number of hippocampal neural progenitor cells [35].

Conversely, some studies have indicated that hippocampus-dependent memory and cognition did not decline or exhibit any observable changes in the context of increased hippocampal neurogenesis. For example, no effect on learning and memory was observed when adult newborn neurons were ablated by X-ray radiation or/and genetic ablation [26]. In contrast with the view that synaptic plasticity is positively correlated with learning ability, cell proliferation increases in the preestrus period, whereas the spatial learning ability decreases [36]. Estrogen improves the survival rate of newborn neurons; however, it impairs spatial learning and memory [37]. Some studies have shown that extremely high levels of cell proliferation may disrupt the neural activity of the hippocampus and interfere with the normal function of the hippocampal dentate gyrus, which is likely due to the lower activation thresholds and higher resting potential of newborn neurons compared with mature neuron synapses [38, 39]. As such, the signal-to-noise ratio of the hippocampal dentate gyrus is reduced, making it difficult to detect excitatory signals [40]. Additionally, accumulating evidence suggests that dysregulation of adult hippocampal neurogenesis may be associated with cognitive decline in neurological disorders and psychological symptoms in psychiatric disorders. Several studies have demonstrated that epileptic patients have an increased number of new neurons in the brain and weakened hippocampus-dependent task performance [41, 42]. These studies indicated that adult-born neurons generated by seizure activity exhibit aberrant cell migration, morphogenesis, and synaptic integration through several signaling pathways and eventually establish recurrent networks. Therefore, seizure-induced enhanced adult neurogenesis substantially reorganizes the local neural network in the dentate gyrus and may impair cognitive functions.

In recent years, although significant progress has been made in the functional study of adult neurogenesis, the understanding of how these diseases interact with adult hippocampal neurogenesis remains very limited. Therefore, although demonstrating the correlations between adult hippocampal neurogenesis and various neuropsychiatric disorders has garnered increasing interest, achieving this goal represents a considerable challenge. One major limitation of studying adult hippocampal neurogenesis in humans is the inability to access live samples. However, some signs of a connection have been elucidated from existing animal models. In these studies, newborn adult neurons would be partly integrated into the neural network to influence neuronal activity; however, the functional significance of adult newborn neurons is not entirely determined by the number of new neurons. Therefore, more in-depth and comprehensive functional studies of adult neurogenesis are required to establish a correlation between the activity of distinct brain circuits, cell types, and cognition.

### 2.2. Methods for the Functional Characterization of Adult Hippocampal Neurogenesis

Hippocampal adult neurogenesis has recently been demonstrated to occur in the adult brain, and it has been proposed to participate in a myriad of behavioral responses, both in physiological states and in the context of neuropsychiatric disorders. However, current studies have not conclusively determined whether adult-born neurons provide important contributions to hippocampal function and, ultimately, how they impact behavior. Disrupting these newborn neurons combined with the evaluation of behavioral changes in animals provides a powerful approach to investigate the function of adult neurogenesis. Common methods for the ablation of newborn neurons include X-ray radiation, genetic ablation, and mitotic inhibitor ablation.

For instance, by utilizing acute or fractionated X-ray irradiation (2 Gy) in mice at postnatal day 21, Peng et al. observed impairments of proliferation and neurogenesis in the subgranular zone of the dentate gyrus but insignificant cognitive dysfunction in subsequent behavioral tests [43]. In another study, using X-ray radiation to inhibit neurogenesis, the relationship between neurogenesis and morphine-induced addiction-related learning and memory was detected [44, 45]. Specifically, the authors reported that reduced neurogenesis caused morphine-related reward memory either to fade or to increase in persistence. These differences in outcomes may be attributed to the morphine dose, the radiation level, and the timing of the detection. Mitotic inhibitors such as cytosine-*β*-D-arabinofuranoside (AraC) could inhibit hypothalamic neurogenesis and could therefore be exploited to observe changes in the behavior of model animals in the context of decreased neurogenesis. Compared to the controls, pups infused with AraC displayed reduced sleep duration, increased sleep interruptions, and a sleep-wake rhythm similar to that of aging mice, favoring the emerging concept that decreased hypothalamic neurogenesis could induce sleep/wake disturbances and senescence-generated sleep disorders [46]. Additionally, genetic ablation is gaining popularity as a tool to characterize the mechanisms of disease onset. Houben et al. evaluated the neurogenesis of tau-expressing disease models and tau-knockout mice and linked tau pathology in the granule cells of the dentate gyrus with the suppression of adult hippocampal neurogenesis, supporting the view that reducing tau expression may promote injury suppression [47]. Given that the role of newborn neurons in the development of chronic epilepsy and related cognitive deficits remains somewhat vague, a study used Nestin-*δ*-HSV-thymidine kinase-EGFP (Nestin-TK) transgenic mice to ablate adult newborn neurons prior to the onset of acute epileptic seizures in mice. The authors found that neuronal ablation decreased the frequency of the seizures, suggesting that abnormal neurogenesis may contribute to epilepsy [41]. The CaM/Tet-DTA transgenic mouse model of hippocampal cell loss has been utilized to examine the impact of neuronal loss on endogenous neurogenesis in aged animals [48]. Moreover, the ablation of neurogenesis regulatory proteins can also reduce neurogenesis. For example, activator protein 2*γ* (AP2*γ*), which exists in a subpopulation of hippocampal transiently expanded progenitor cells, acts as a modulator of adult hippocampal glutamatergic neurogenesis in mice. The ablation of activator protein 2*γ* can significantly reduce hippocampal neurogenesis and disrupt neural continuity between the ventral hippocampus and the medial frontal cortex [49].

**Figure 2:**
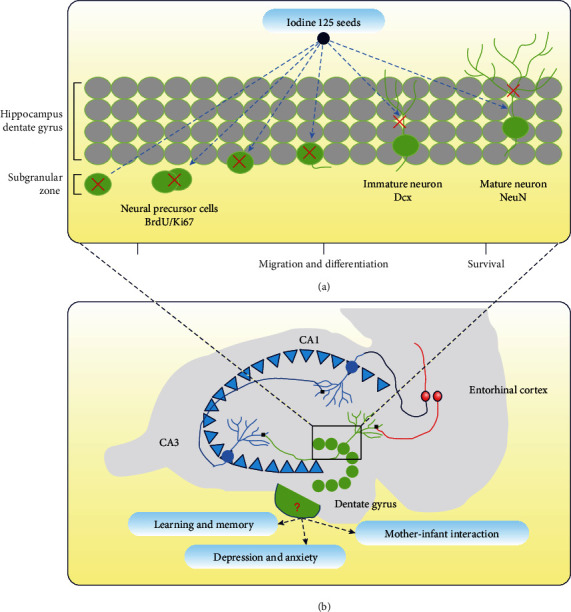
Schematic diagram of ^125^I brachytherapy for ablation of hippocampal neonatal neurons (a), functional changes in hippocampus (b). Red crosses in (a) indicate ablation of newborn neurons.

Some studies have indicated that various strategies to ablate neurogenesis produce a limited impairment in some hippocampal-dependent learning and memory tasks. Moreover, due to the lack of spatial and cellular specificity of most ablation techniques, it is difficult to ascertain whether the consequent behavioral effects were caused by ablation of neurogenesis or other impairments [50]. To circumvent these problems, our group was the first to utilize iodine-125 seed brachytherapy for neuron ablation in the hippocampus (Figure 2). This procedure not only is simpler and safer than conventional X-ray ablation but also has greater spatial specificity and produces less trauma than other mitotic inhibitors and genetic neuron ablation. We not only confirmed that ^125^I brachytherapy could ablate newborn neurons in the hippocampus but also evaluated its effect on the ablation of hippocampal adult neurogenesis under different doses, time, and space (unpublished work).

## 3. Effect of Reproduction-Associated Hormones on Adult Hippocampal Neurogenesis

### 3.1. Estrogen

#### 3.1.1. Distribution of Estrogen and Its Receptors in the Brain

Estrogen is involved in numerous physiological processes including the development and maturity of follicles and female sex organs, the formation and differentiation of breast acini and milk production, the maintenance of female secondary sexual characteristics, the movement and maturation of eggs in the fallopian tube, and the metabolism of the endometrium and smooth muscle. Estrogen is in fact a group of female sex hormones, which encompasses three major endogenous hormones: estrone, estriol, and estradiol [1]. Estrogen is mainly synthesized by the corpus luteum of the ovary and the fetal placental unit. Its levels increase continuously from the onset of pregnancy until just prior to labor, after which they decrease sharply and remain low during lactation [51].

Estrogen receptors are comprised of nuclear estrogen receptors (ER*α* and ER*β*) and membrane estrogen receptors (GPER1, ER-X, and Gq-mER) [52]. ER*α* (with a molecular weight of 67 kDa) and ER*β* (with a molecular weight of 60 kDa) are located in the cytoplasm when inactive and are distributed throughout the brain in the hippocampus, hypothalamus, amygdala, and preoptic area [53]. GPER1, a membrane-bound G protein-coupled estrogen receptor mainly located on the plasma membrane, was originally identified in the endoplasmic reticulum of neurons and was found to be ubiquitous in various cortical areas including the hippocampus, olfactory bulb, hypothalamus, motor and somatosensory centers, piriform cortex, and the nucleus of the epithalamus, particularly in the CA3 area of female rats [54, 55]. Unlike ER*α* and ER*β*, GPER1 mainly mediates estrogen to activate MAPK/ERK and other signaling pathways [52]. ER-X (with a molecular weight of 63 kDa), which was detected during research on the development of the cerebral neocortex, shows high affinity to the isomers of E2, 17*α*-E2, and 17*β*-E2 compared with other estrogen receptors [56]. Gq-mER was first discovered in the arcuate nucleus of the hypothalamus and promoted estrogenic activity associated with hypothalamic reproduction and energy homeostasis [52, 57]. So far, ER-X and Gq-mER have only been reported to be distributed in the cerebral cortex and the hypothalamus.

Estrogens (estradiol and estrone) regulate multiple cellular processes including cell proliferation and survival, which underlies their impact on neurogenesis [31, 60, 61]. Their regulatory effects are related to the time and dosage of the estrogen treatment, as well as the age and sex of the subjects (Table 1). Despite many reports on the effect of estrogen on adult hippocampal neurogenesis in female mammals, data on its effect on male mammals are scarce [64, 68]. However, the available data has indicated that estradiol has a slight influence on hippocampal neurogenesis in males. The sections below provide an overview of the correlation between endogenous and exogenous estrogens and adult hippocampal neurogenesis.

#### 3.1.2. Endogenous Dynamic Estrogen and Adult Hippocampal Neurogenesis

Natural fluctuations in ovarian hormones persist throughout the female lifecycle and are particularly visible in women's menstrual cycle, reproductive stage, and aging transition.

The estrous cycle of mammals is divided into four stages: proestrus, estrus, late estrus, and diestrus. Hormone level variations exist at each stage. In the proestrus period, serum estrogen and progesterone levels increase and peak, whereas they decrease in the diestrus period [69]. Hormone levels can affect the proliferation of neurons in the dentate gyrus of the hippocampus, which in turn influences adult hippocampal neurogenesis. During the proestrus period, increased cell proliferation was observed in the hippocampal dentate gyrus of rats [58, 70, 71], whereas the cell proliferation rate decreased during the diestrus. Conversely, studies in mice showed only a minor change in cell proliferation in each estrus stage [70], which was likely because the ovarian hormones only fluctuated for a few hours and the fluctuation was not captured. Additionally, some studies have characterized the seasonal and nonseasonal breeding of prairie voles and have demonstrated that estrogens have different effects on neurogenesis at different periods in their lifecycle [72].

Diminished estrogen secretion is strongly correlated with decreased gonadal function in female menopause [73]. In aging female rats, the estrus cycle becomes irregular and prolonged from midlife onwards and eventually reaches the noncirculatory stage of a continuous estrus interval where the proliferation and survival rates of neurons decrease [74, 75], indicating that the decline in neurogenesis in aging rats is related to the natural reduction in estrogen secretion. The influence of endogenous ovarian hormone fluctuations on cell proliferation is highly debated, and studies on the influence of endogenous ovarian hormone fluctuations on newborn neuron survival are scarce.

Taken together, current findings indicate that endogenous estrogen could promote the proliferation of hippocampal dentate gyrus nerve cells. However, in-depth research on the effect of single-estrogen treatment on neurogenesis is needed to eliminate the potential confounding effects of other hormones.

#### 3.1.3. Exogenous Estrogen and Adult Hippocampal Neurogenesis

*(1) Effect of Estrogen on the Proliferation of Hippocampal Dentate Gyrus Cells*. Adult bilateral ovariectomized (OVX) animal models were used to investigate the effects of estrogen on neurogenesis. Compared with the sham operation group, the serum estradiol level in the short-term OVX rats decreased sharply within 24 hours, with a concomitant decline in cell proliferation and the number of newly born immature neurons [31, 76], which could be reversed by acute exposure to estrogens [58, 59]. In contrast, no observable change in neuronal proliferation was detected after long-term estrogen deprivation (4 weeks) in rats and mice by ovariectomy and subsequent high-dose estrogen treatment [59], possibly owing to the presence of locally synthesized estrogen in the hippocampus leading to the resumption of cell proliferation. There is evidence that topical estradiol application affects hippocampal cell proliferation in 5-day-old rats [77]; however, several studies in which little or no estradiol was detected in the hippocampus of adult rats 2-3 weeks after ovariectomy deny this hypothesis. Goel et al. indicated that the decline of estrogen, which could stimulate the hypothalamic-pituitary-adrenal axis, may lead to a decrease in cortisol. This in turn inhibits cell proliferation, resulting in an increased proliferation of neural cells [78], and therefore, the limited alteration in the cell proliferation rate observed after long-term estrogen deprivation might have been due to the involvement of cortisol [79]. Additionally, the serotonin in the hippocampus may also have important effects [80]. Cell proliferation was unaffected after long-term estrogen deprivation and subsequent short-term high-dose estrogen treatment, which was likely due to the decreased sensitivity to estrogen in the neuron precursor cells.

Further, the duration of estrogen exposure also plays a regulatory role in hormone activity. Seven days after bilateral ovariectomy, estradiol and estrone (10 *μ*g) could increase the cell proliferation rate after exposure for 30 minutes and 2 h, whereas the cell proliferation rate slightly increased after exposure to estradiol benzoic acid (EB) for 4 h [58, 59, 61, 63]. Ormerod et al. found that cell proliferation increased in female prairie voles after acute EB administration for 4 hours but was inhibited 48 hours later [60]. Seven days after bilateral ovariectomy, rats were exposed to EB for 20 days. Subsequently, they were injected with BrdU for 2 hours and showed no change in proliferation. The same result was reported under long-term exposure to various estrogens [31, 65, 66]. As previously noted, the inhibition of cell proliferation is mediated by the stimulation by estrogen with adrenal steroids. Zhang et al. established a hormone-simulated pregnancy (HSP) mouse model. Seven days after bilateral ovariectomy, the HSP postpartum estrogen nonwithdrawal group and the postpartum EW group were exposed to both EB (0.5 *μ*g/day) and progesterone (0.8 mg/day) for 16 days [31]. The EW group was treated with EB for 7 consecutive days, and samples were taken after 5 days of survival (day 28). In contrast, the HSP group was treated with EB for 12 consecutive days (day 28). The expression of S_28d_BrdU^+^ was downregulated in the EW group compared to the control, whereas the expression of S_10d_BrdU^+^ remained unchanged. The expression of both S_28d_BrdU^+^ and S_10d_BrdU^+^ in the HSP group was upregulated, indicating that postpartum EW could impair hippocampal neurogenesis in mice. We observed that the rapid withdrawal of estrogen may lead to the massive death of newborn neural progenitor cells. In other cases, estradiol could stimulate cortisol secretion, thereby restraining cell proliferation. In summary, short-term exogenous exposure to estrogen stimulates the proliferation of neurons, whereas long-term exposure has negligible or even inhibitory effects.

In addition to exposure duration, estrogen species is another factor that influences neuronal proliferation. In female rats, proliferative activity increased after exposure to 17*α*-estradiol, 17*β*-estradiol, and estrone for 30 minutes but remained constant after exposure to EB, which was likely due to the slower metabolism of EB in comparison to other estrogens [61, 81].

Additionally, the effect of estrogen on the proliferation of neurons is dose-dependent. An injection of 0.3 *μ*g or 10 *μ*g of estradiol is equivalent to the circulating serum levels of estradiol in the diestrus and proestrus stages, respectively [82]. This treatment could decrease cell proliferation rates after ovariectomy, thus reverting them to their preovariectomy rate [61]. In contrast, a medium dose of 1 *μ*g or a high dose of 50 *μ*g was generally ineffective in restoring the cell proliferation rate [59, 61].

*(2)Effect of Estrogen on the Survival of Hippocampal Dentate Gyrus Cells*. Neural cell proliferation refers to the production of newborn neurons, whereas neuronal survival emphasizes the long-term process by which neurons are generated, differentiate, and mature. However, most studies have thus far focused primarily on neuron proliferation, whereas neuron survival has remained largely understudied. Neuronal survival was reduced in OVX rats that were bilaterally ovariectomized (day 0), labeled with BrdU (day 6), exposed to EB (day 7) for 16 consecutive days, and then perfused [64]. The same conclusion was reached after 22 days of continuous exposure to EB [66]. In another study, 7 days after bilateral oophorectomy, rats were exposed to estradiol or estrone for 19 consecutive days and BrdU was injected after 24 hours of hormone exposure [65]. The results showed a diminished proliferation rate in rats exposed to estrone, which was likely due to the difficulty of surviving in an estrone-rich environment [83]. Additionally, Zhang et al. reported that the survival rate of neurons rose in OVX mice exposed chronically to EB and progesterone but decreased in an estradiol retreatment group [31]. It is worth noting that the hormone affected both neuron proliferation and survival after estrogen exposure, whereas it only affected the survival of neurons produced prior to hormone exposure. In summary, the survival of newborn neurons is inhibited before estradiol treatment but enhanced thereafter due to an increased proliferation rate [64–66], showing that an estradiol-rich environment is ideal for the survival of neurons produced under the stimulation of this hormone. This was further validated by the decline in neuronal survival upon rapid postpartum estradiol retreatment in HSP. Additionally, prior behavioral tests or spatial training of subjects may interfere with the proliferation and survival of nerve cells, which should be taken into consideration when designing future experiments [65, 67].

### 3.2. Progesterone

#### 3.2.1. Distribution of Progesterone and Its Receptors in the Brain

Progesterone is a reproduction-associated steroid hormone that is mainly produced by the corpus luteum in the first 8 weeks of pregnancy and secreted by the placenta at 8-12 weeks. Importantly, this hormone can coordinate multiple estrogen-mediated physiological and behavioral processes. During pregnancy, the progesterone level rises continuously, peaks within 4 weeks before delivery, and rapidly decreases after delivery or in the third trimester [69, 84]. Progesterone regulates the smooth muscle of the uterus, reduces its sensitivity to OT, and ensures the normal growth and development of fertilized eggs [1]. PRA and PRB are representative progesterone nuclear receptors, which are broadly distributed in several brain regions including the hippocampus, the frontal cortex, the hypothalamus, the cerebellum, the medial inner side of the amygdala, and the bed nucleus of the stria terminalis [85]. Another class of progesterone receptors called membrane progesterone receptors (mPRs) include mPR*α*, mPR*β*, and mPR*γ* [86, 87], among which mPR*α* is the most pharmacologically active and enriched in the olfactory bulb, cortex, hypothalamus, hippocampus, and cerebellum. In contrast, mPR*β* is mainly distributed in the hippocampus, hypothalamus, midbrain, and forebrain of mammalian brains [88]. Additionally, the b5-like heme/steroid binding protein family includes progesterone membrane receptor module 1 (PGMRC1) [89], which is distributed in the forebrain and regulates neuroendocrine function, as well as the hippocampus, cortex, and cerebellum [90].

#### 3.2.2. Progesterone and Adult Hippocampal Neurogenesis

Progesterone regulates spinal cord production, synaptogenesis, neuron survival, and dendritic growth and plays a neuroprotective role in animal models of neurodegenerative diseases and acute brain damage [85, 91]. However, few studies have thus far characterized the effects of progesterone on the proliferation and survival of nerve cells. Previous studies have revealed that in vitro treatment with progesterone may increase the proliferation of hippocampal nerve cells in female rats in a dose-dependent manner [92]. The same is true for tetrahydroprogesterone, a progesterone metabolite [93]. Additionally, exposure to progesterone (4 mg/kg) for 1 hour could increase cell proliferation in the granulosa cell layer of the hippocampal dentate gyrus in OVX rats [90, 93]. However, with prolonged exposure to progesterone (chronic treatment), cell proliferation and survival remained constant in OVX rats, and female OVX rats showed almost no change in neurogenesis after long-term administration (for 21 days) of progesterone (1 or 4 mg/kg) or medroxyprogesterone acetate (MPA) [66]. Brinton et al. found that exposing 18-day-old rat embryos to progesterone alone for 24 hours could induce a 20% increase in rat hippocampal neurons [87], suggesting that treatment with progesterone alone may promote the proliferation of hippocampal neural progenitor cells similar to tetrahydroprogesterone. Zhang et al. exposed mice to EB and progesterone for 17 consecutive days, treated them with EB alone for 5 days to establish an HSP model [31] (Table 1), and found that the number of BrdU+ cells did not decrease in the HSP mice, indicating that progesterone withdrawal did not lead to the death of immature newborn neurons. Instead, EB treatment might diminish the number of mature newborn neurons in the brain of mice in the HSP postpartum EW group, which remains to be confirmed. Additionally, treating male rats with progesterone for 7 consecutive days could increase the cell proliferation in the dentate gyrus of the hippocampus but had no detectable effect on the survival rate [94]. Male mice were exposed to progesterone for 3, 7, 28, and 56 days, but the survival rate of hippocampal dentate gyrus cells only increased after 3 days of treatment with progesterone, indicating that progesterone increased the survival rate of relatively young newborn neurons [95].

During the menstrual period, estrus cycle, and reproductive period, progesterone and estrogen exhibited similar dynamic changes. Given that progesterone mediates the effect of estrogen on neurogenesis, scholars prefer to use estrogen and progesterone in combination to observe changes in neurogenesis. Tanapat et al. inhibited the estradiol-induced proliferation of hippocampus dentate gyrus cells in adult female ovariectomized rats through 2 injections of estradiol (10 *μ*g each) with an interval of 2 hours, followed by progesterone treatment after 48 hours [59]. Progesterone and BrdU were injected two days after the female rats were injected with 17*β*-estradiol or EB (10 *μ*g), which inhibited estradiol-induced cell proliferation [59, 90]. When estradiol and progesterone were injected simultaneously into the OVX rats, the increase in cell proliferation induced by estradiol was not blocked but was lower than the level of cell proliferation induced by the injection of estradiol alone [93]. Although chronic MPA treatment had no observable effect on the neurogenesis of the female hippocampal dentate gyrus, the injection of MPA combined with EB (10 *μ*g) for 21 days reduced the cell survival rate of adult female rats [66]. The proliferation of hippocampal cells in the OVX rats increased after exposure to a high estradiol dose but was inhibited upon exposure to a progesterone antagonist. Simultaneous acute exposure to estradiol and progesterone would not lead to synergistic effects [93]. Importantly, progesterone alone could enhance the proliferation of nerve cells, while its combination with other sex hormones would have an inhibitory effect [59, 92].

In the context of cerebral ischemia and acute brain injury, not only could progesterone inhibit brain injury-induced cell proliferation in male rats, but it also enhanced the survival of newborn neurons inhibited by ischemia [96]. Additionally, progesterone treatment after traumatic brain injury could restore cell proliferation and cell death to normal levels in the dentate gyrus of the hippocampus [94].

The use of progesterone alone has few negative effects on neurogenesis, whereas it may serve as a suppressor when combined with other hormones. Regardless, the effects of progesterone (alone or combined with other hormones) on neurogenesis seem to correlate with the concentration of progesterone, the sex of the subject, and the hormone treatment time; however, this remains partly ambiguous and requires further investigation.

### 3.3. Prolactin (PRL)

#### 3.3.1. Distribution of PRL and Its Receptors in the Brain

PRL belongs to a hormone family that includes growth hormone and placental PRL and is synthesized and secreted by anterior pituitary cells as well as extrapituitary regions such as the breast, placenta, and uterus [97]. This pleiotropic pituitary hormone is known to have more than 300 physiological effects and has a regulatory role in reproduction, immune regulation, angiogenesis, energy metabolism, osmotic balance, and development [98, 99]. For instance, PRL could promote the proliferation of breast cells, stimulate lactation and the synthesis of steroid hormones, accelerate bone metabolism, and regulate the composition of amniotic fluid and osmotic pressure in the uterus. It also plays a fundamental role in promoting the secretion of progesterone and the maturing of follicles [100]. The PRL level increases early in pregnancy, rises rapidly just before and immediately after delivery, and remains high under the stimulation of breastfeeding [101].

PRL receptors exist in many nuclei and subregions of the hippocampus, SVZ, ventricular choroid plexus, and hypothalamus (including the anterior medial nucleus, ventrolateral preoptic nucleus, supraoptic nucleus, paraventricular nucleus, periventricular nucleus, dorsal medial hypothalamic nucleus, ventromedial hypothalamus, and arcuate nucleus) [102, 103]. Moreover, the expression level of PRL receptor genes changes during different periods. For instance, it is significantly upregulated during pregnancy and lactation [104].

#### 3.3.2. PRL and Adult Hippocampal Neurogenesis

PRL plays numerous roles in the central nervous system. To cite a few examples, this hormone induces parenting behaviors in females, stimulates OT neurons, promotes appetite and food intake, facilitates the secretion of ACTH in response to stress, inhibits fertility, and protects neurogenesis under stress [105–108].

PRL is generally thought to serve as a neurogenesis activator in SVZ neurogenesis [100, 109]. However, little correlation was initially observed between PRL and hippocampal neurogenesis. On the 7th day of pregnancy and the 7th day after delivery (under stress), PRL could enhance the proliferation of SVZ cells but had little effect on the proliferation of hippocampal dentate gyrus cells [110], which was possibly due to the method of PRL administration or dose. Later, Lajud et al. found that injecting PRL into rats for 14 consecutive days at the immediate postnatal stage could reduce the neurogenesis of the dentate gyrus and olfactory bulb and aggravate the depressive state of adult male and female rats, thus demonstrating that the effect of PRL on neurogenesis depends on the age of the animal and that PRL is involved in mood regulation [111]. Conversely, other authors have proposed that PRL plays a protective role in adult hippocampal neurogenesis. Torner et al. demonstrated that PRL not only protected hippocampal neurogenesis from stress or glucocorticoids by acting as a regulator of brain cell proliferation but also could perform its functions without perturbing the cell proliferation of the hippocampal dentate gyrus under stress-free conditions [102, 112]. PRL counteracts the adverse effects of glucocorticoids on hippocampal neurogenesis through PRL receptor-mediated mechanisms, and this might be the mechanism by which PRL protects adult neurogenesis during chronic stress [102]. Mak et al. demonstrated that PRL injection into the lateral ventricle of male mice could increase the proliferation of neuron precursor cells in the dentate gyrus and the PRL signal may be associated with parent identification and enhanced neurogenesis in the olfactory bulb and hippocampus [113]. Together with the findings of Wagner et al., the aforementioned results suggested that PRL affects hippocampal neurogenesis through indirect mechanisms [114]. Walker et al. extended prior research and found that exogenous PRL treatment could increase the number of hippocampal dentate gyrus neuron precursor cells both *in vivo* and *in vitro* [115]. Compared with the control, PRL gene-deficient mice exhibited a reduced number of hippocampal neuron precursor cells, which impaired hippocampal-dependent learning and memory, confirming that PRL could promote the division of neuron precursor cells in the hippocampal dentate gyrus.

PRL binds to its receptors in the hippocampus to regulate neurogenesis [102, 115]. Most studies have demonstrated that PRL either promotes or has no effect on hippocampal neurogenesis, and only few studies have indicated that PRL functions as an inhibitor. These contradictions may be due to differences in the PRL dose, the age of the subject, and the route of administration, among other factors. Overall, these findings provide preliminary evidence that PRL promotes hippocampal neurogenesis to some extent and influences mood disorders such as depression and anxiety.

### 3.4. Human Chorionic Gonadotropin (hCG)

hCG, a glycoprotein secreted by the trophoblast cells of the placenta, is comprised of *α* and *β* dimers, of which the *α* subunit is similar to the follicle-stimulating hormone (FSH), luteinizing hormone (LH), and thyroid-stimulating hormone (TSH) secreted by the pituitary. Therefore, these hormones can cross-react with each other, whereas the structure of the *β* subunits is apparently divergent [1, 116]. In the nonpregnant state, hCG is produced by the pituitary gland and plays a crucial role in the establishment, promotion, and maintenance of human pregnancy [117]. For instance, hCG could promote the production of progesterone, cytotrophoblast cell differentiation, placental and uterine endothelial angiogenesis, the formation and differentiation of fetal organs, and the growth and development of the umbilical cord, in addition to maintaining the quiescence of the myometrium and the growth of the uterus in line with that of the fetus [116]. Additionally, hCG orchestrates the immune response *in vivo* and serves as a biomarker for a variety of tumors [116]. During pregnancy, there is a sharp increase in hCG levels in the first trimester, which peaks at approximately 10 weeks of gestation, slowly decreases until the 20th week, and then remains stable until the end of pregnancy [1]. Its specific receptors are distributed in the hippocampus, dentate gyrus, hypothalamus, cortex, brainstem, posterior fossa, cerebellum, choroid plexus, ependymal cells, glial cells, neural retina, pituitary gland, and spinal cord of adult mammals [118]. Nevertheless, few studies have assessed the effects of hCG on the neurogenesis of the adult hippocampus.

### 3.5. Relaxin

Relaxin, a small peptide hormone belonging to the insulin/relaxin family, is mainly produced in the corpus luteum in the ovaries of pregnant mammals [119]. It was named after its relaxing and lengthening effect on the pubic symphysis during guinea pig delivery. As a vasodilator in mammalian delivery, relaxin facilitates ligament relaxation and birth canal expansion before delivery, adjusts the hemodynamic changes in both the nonpregnant state and the pregnant state, regulates the secretion of OT and antidiuretic hormone, promotes the development of mammary glands, and regulates the lactation reflex and plasma osmotic pressure. Additionally, this hormone is involved in the establishment of pregnancy and adjusts the mother's cardiovascular system to support pregnancy and the maternal-fetal interface in preparation for postpartum breastfeeding [120]. It also has antifibrosis, organ protection, and antiallergic effects [121, 122].

The relaxin peptide family consists of relaxin receptor 1, receptor 2, receptor 3, and other relaxin family peptide receptors [123, 124] whose cell signals are mediated through their corresponding homologous G protein-coupled receptors [125]. The circulating concentration of relaxin begins to rise in the first trimester, peaks at approximately the 12th week of pregnancy, then decreases until roughly the 17th week of pregnancy, remains constant during the rest of the pregnancy, and decreases sharply after delivery [126].

Relaxin plays an important role in memory consolidation and motor activities and is involved in somatosensory and autonomic endocrine pathways [127, 128]. Notably, relaxin 3, the most highly expressed relaxin peptide family member preferentially distributed in the midline cap layer near the fourth ventricle [129, 130], was confirmed to have pivotal biological functions in both reproduction and central nervous system [131]. Its receptor, GPCR135, is expressed in the hippocampus, septum, intergenic lobules, fourth ventricle, and amygdala [128, 132]. Relaxin 3 has been proposed to function as a mediator of emotions and behavior due to its role in activating arousal and behavior, as well as regulating appetite, the stress response, anxiety, memory, sleep, and circadian rhythms [130, 133]. Silvertown et al. demonstrated that relaxin 3 derived from macaques has a neuroprotective effect on human neuronal cell cultures [128]. Few reports have described the correlation between relaxin and neurogenesis. In light of the above-described relaxin 3-mediated activities in the central nervous system, future studies should determine whether and how relaxin 3 mediates neurogenesis in the adult hippocampus dentate gyrus.

### 3.6. Glucocorticoids

#### 3.6.1. Distribution of Glucocorticoids and Their Receptors in the Brain

Glucocorticoids are a type of steroid hormone secreted by the fascicular zone in the adrenal cortex, which have strong effects on the metabolism of carbohydrates; regulate the biosynthesis and metabolism of sugar, fats, and proteins; suppress the immune response; and dampen inflammatory responses, in addition to exhibiting antitoxic and antishock behavior. The serum level of glucocorticoids in rats decreases slightly in the first trimester, rises sharply from the second trimester, peaks in the third trimester, and finally decreases to nonpregnancy levels after delivery [51]. Glucocorticoid receptors are located in the hippocampus, striatum, amygdala, forebrain, paraventricular nucleus of the hypothalamus, preoptic area, ventromedial nucleus of the thalamus, the periventricular zone of the optic tectum, and the pituitary gland [134, 135].

#### 3.6.2. Glucocorticoids and Adult Hippocampal Neurogenesis

High levels of corticosterone could inhibit the proliferation of hippocampal neuron precursor cells and the survival of neurons [136]. Brummelte and Galea found that high levels of glucocorticoids inhibited hippocampal neurogenesis in both male and female rats [79]. Torner et al. found that the neurogenesis in the hippocampus dentate gyrus of mice was significantly diminished in cases of high glucocorticoid levels induced by chronic stress, the extent to which was closely correlated with the glucocorticoid level [102]. Studies in rats, tree shrews, and marmosets have confirmed these findings [137–139].

Apart from the aforementioned studies on the effect of glucocorticoid levels on neurogenesis in the nonpregnant state, scholars have also investigated the effect of glucocorticoid levels on neurogenesis in the reproductive state due to the influence of reproduction on the hypothalamus-pituitary-adrenal axis and hippocampal plasticity [136, 140]. In light of the high sensitivity of the maternal brain to high concentrations of glucocorticoids during pregnancy and postpartum, elevated levels of glucocorticoids induced by stress would inhibit neurogenesis in the hippocampal dentate gyrus of rats. Leuner and Sabihi observed a decrease in this hormone and a subsequent reduction in the blockage of cell proliferation by virtue of adrenalectomy or evacuation of pups during childbirth, further confirming that high glucocorticoid levels have an inhibitory effect on hippocampal neurogenesis [141].

### 3.7. Oxytocin (OT)

#### 3.7.1. Distribution of OT and Its Receptors in the Brain

OT is a brain pituitary neurohormone secreted by giant cells in the supraoptic nucleus and paraventricular nucleus of the hypothalamus, which is transported to the posterior pituitary through the nerve fibers of the hypothalamic-pituitary axis [142]. This hormone can be projected along axons of hypothalamic neurons to diverse areas, such as the amygdala, hippocampus, striatum, suprachiasmatic nucleus, terminal striatal nucleus, and brainstem, and acts as a neuromodulator or neurotransmitter, thereby affecting neurotransmission in these areas [143]. In addition to its release in the axonal terminal, OT could be released into the extracellular space via dendrites, and therefore, its effects are not only local but also spread through the brain to reach distant targets. Some peripheral tissues such as the uterus, corpus luteum, amniotic membrane, and placenta can also synthesize OT [144]. This hormone is commonly used clinically to induce labor, maintain postpartum homeostasis, and shorten the third stage of labor. OT also stimulates lactation, promotes milk ejection, induces gravid uterine contraction, and reduces the level of stress hormones such as corticosterone to lower blood pressure [107, 144–146]. Regarded as an initiator of intimate relationships, OT has been confirmed to regulate maternal behavior to enhance self-confidence and establish cooperation [142, 147]. OT neurons have extensive projections throughout the brain regions including the medial preoptic area, the bed nucleus of the stria terminalis, the ventral tegmental area, the ventral striatum, and the amygdala, all of which are critical areas of maternal behavior [143, 148]. The functional level of OT is negatively correlated with postpartum depression and anxiety [149].

During pregnancy, serum OT concentration may vary widely among individuals depending on emotions and the mother-infant relationship. The serum OT level, which gradually rises throughout pregnancy, rises substantially in the third trimester and after delivery due to stimulation from mother-infant interactions [1, 150]. From the third trimester to postpartum, the basal activity of OT receptors and OT-energetic cells increases with the strengthening of the mother-infant relationship [151]. The OT signal is mediated by the OT receptor (OTR), which belongs to the G protein-coupled receptor family and exists in the prefrontal cortex, the preoptic area, the lateral septum, the hippocampus, the hypothalamus, and the medial amygdala of mammals [152–154].

#### 3.7.2. OT and Adult Hippocampal Neurogenesis

Little is known regarding the correlation between OT and adult neurogenesis. Existing studies have shown that exogenous OT could stimulate hippocampal cell proliferation and neurogenesis in rats. Further, OT could prevent the inhibitory effect of stress hormones on hippocampal neurogenesis and enhance the proliferation of newborn neurons in rats under cold water swimming stress [155]. Opendak et al. proposed that *in vitro* OT treatment could stimulate the neurogenesis of the dentate gyrus in adult male rats [156]. Taken together, these observations indicate that OT promotes the proliferation of nerve cells; however, its influence on hippocampal dentate gyrus neurogenesis in pregnant mammals remains ambiguous. Given the neurological effects of OT in mediating maternal nurturing behavior, cognition, and mood disorders, the effect of OT on the neurogenesis of the hippocampal dentate gyrus in the reproductive state is worth studying in more detail.

## 4. Potential Mechanisms Underlying the Role of Hormones on Adult Hippocampal Neurogenesis Taking Estrogens as an Example

### 4.1. Receptors

Researches have proposed that the effects of estrogen on hippocampal neurons are partly mediated by estrogen receptors [62, 157] (which encompass ER*α*, ER*β*, and GPER in the hippocampus as discussed in previous sections), with the regulation of cell proliferation being the most affected. A series of experiments was performed using estradiol receptor agonists and inhibitors, which suggested that estradiol receptor agonists promoted the proliferation of nerve cells [62]. In contrast, estrogen inhibitors reversed the upregulation of cell proliferation induced by estradiol [158], resulting in the coexpression of estrogen receptors in neural progenitor cells in the granular cell layer of the dentate gyrus [62], thus confirming the receptor-mediated effect by which estrogen affected hippocampal neurogenesis. Genomic and nongenomic mechanisms have been proposed to explain the regulatory effects of estrogen on neurogenesis.

#### 4.1.1. Genomic Mechanism (Figure 3)

Estrogen binds to its receptor, ER*α* or ER*β*, which is distributed in the cytoplasm or nucleus, resulting in the formation of a homodimer or heterodimer. It is then transported to the cell nucleus and binds to estrogen response elements (EREs) located in the target promoter region on the DNA to regulate gene expression and protein synthesis as a transcription factor. Given that some estrogen-regulated genes do not contain ERE sequences [159], gene expression is regulated through mechanisms involving other transcription factors and corresponding response elements, such as protein-protein interactions [159, 160].

#### 4.1.2. Nongenomic Mechanism (Figure 3)

In addition to the genomic mechanism in which estrogen binds to nuclear receptors, nongenomic effects in the hippocampus are mediated by estrogen receptors in the plasma membrane (extranuclear receptors) [161]. This process is rapid, occurring in a few seconds or minutes, and transient (1-4 hours) [56]. For instance, enzymes through which estrogens regulate cell signaling associated with hippocampal memory were rapidly phosphorylated by multiple membrane receptors on the dorsal side of the hippocampus, including metabotropic glutamate receptor (mGluR) 1a and its interaction with ER*α* and ER*β*, N-methyl-D-aspartate (NMDA) receptor [162], and G protein-coupled estrogen receptor (GPER) [163]. The activation of cell signaling pathways may trigger epigenetic changes such as histone acetylation, thereby regulating gene transcription and protein synthesis. Alterations in cell signal transduction may also affect the local protein levels of internal structures such as dendritic spines.

### 4.2. Neuroimmune Mechanisms

Beyond receptor mechanisms, other factors may influence the onset of adult hippocampal neurogenesis [19, 65]. For instance, microglia, the innate immune cells in the brain, provide support for adult neurogenesis under physiological conditions in the hippocampus [164, 165]. The majority of newborn cells in the hippocampus cannot survive to maturity [166]. Instead, most cells undergo apoptosis and microglial phagocytosis within the first 1-4 days of birth. Additionally, microglia are the predominant mediator of neuroinflammation, which has been found to correlate with the regulation of adult hippocampal neurogenesis based on accumulating evidence [167]. The nervous system becomes populated with activated microglia in cases of pathological inflammation, which is accompanied by a decreased newborn neuron survival rate, whereas the proliferation of nerve cells remains unaffected. However, microglia inhibitors could restore the survival rate of neurons [165]. The anti-inflammatory effects of estrogen on the brain have been broadly documented in central nervous system diseases, including stroke and neurodegenerative diseases [168–171]. In vitro studies have confirmed that estrogens significantly affected the microglia function of rat primary microglia cell cultures or the N9 microglia cell line. For example, estradiol could suppress lipopolysaccharide-induced microglial activation [172]. Given the role of inflammation and microglia in neurogenesis, as well as the anti-inflammatory effects of estrogen, it is conceivable that the effect of estrogen on neurogenesis is partly achieved by regulating microglial inflammatory processes. Further, estrogen-deficient microenvironments may lead to immune dysregulation and perturbed neurogenesis. However, to the best of our knowledge, a direct link between estrogens and neurogenesis has not been established.

### 4.3. Brain-Derived Neurotrophic Factor

Brain-derived neurotrophic factor (BDNF) is an influential neurotrophin during neurogenesis and binds to a membrane receptor, TrkB, mediating the survival, growth, and maintenance of neurons in key brain circuits that contribute to emotion and cognitive functions. Therefore, alterations in the BDNF level generally typically affect neurogenesis rates [173, 174]. Many studies have indicated that BDNF has a critical role in neurogenesis [175, 176]. For example, BDNF plays an important role in regulating the basal level of neurogenesis in the dentate gyrus of adult mice, reversing dietary restriction-induced decreases in neurogenesis by enhancing the survival of newborn neurons. Sairanen et al. proposed that BDNF signaling was required for the long-term survival of mouse hippocampal neonatal neurons. Moreover, rising BDNF levels in the dentate gyrus coincided with an increase in hippocampal dentate gyrus neurogenesis [177]. Decreases in BDNF levels associated with depression are thought to be one of the main pathological mechanisms underlying impaired hippocampal neurogenesis [178]. Additionally, given that the effects of estrogen paralleled those of BDNF [179], further studies demonstrated that estrogens directly regulated BDNF, including ERE recognition on the BDNF gene. For instance, BDNF mRNA levels were significantly reduced after bilateral ovariectomy in rodents, whereas the opposite outcome was observed after estradiol treatment [180, 181]. The activation of estrogen receptors selectively reinforced BDNF signaling and BDNF expression, thereby promoting hippocampal dentate gyrus neurogenesis [182, 183]. The expression of BDNF was enhanced in the hippocampus of HSP mice and OVX/EB mice but decreased in the EW and OVX groups [31]. Therefore, we hypothesize that the effect of estrogen on adult hippocampal neurogenesis is largely mediated by the regulation of BDNF.

## 5. Conclusions and Future Perspectives

The effect of reproduction-associated hormones on the renewal of adult hippocampal neurons varies with hormone dose, the time of administration, experimental treatment, and subject gender and age, among other factors. Short-term estrogen exposure stimulated the proliferation of nerve cells, whereas long-term treatment may have the opposite effect. Progesterone alone has no inhibitory effect on neurogenesis but reduces the effects of other hormones on neurogenesis when combined with other hormones. Generally, short-term estrogen exposure, progesterone treatment alone, and PRL and OT activity have a positive effect on adult hippocampal neurogenesis, whereas glucocorticoids may have inhibitory effects. Moreover, little research has been conducted on the effects of relaxin or hCG.

Most current studies have focused on the effects of multiple reproductive hormones in isolation. Further, except for the combination of estrogen and progesterone, research on the combined effects of multiple hormones on neurogenesis is scarce. Hormones change constantly *in vivo* and show complex interactions during pregnancy and postpartum. Given that the impact of these hormones on neurogenesis and the functional significance of the newborn neurons, it is hoped that more research will be conducted in these areas.

Although there have been tremendous advances in the understanding of adult hippocampal neurogenesis in the past few years, few studies have assessed whether the neonatal neurons in the hippocampus are activated and what influence they have on spatial learning and memory, particularly in reproductive state mammals, which are likely to exhibit a low proportion of activated newborn neurons. Therefore, future studies should focus on establishing a causal relationship between reproduction-related hormones and brain plasticity, in addition to determining whether newborn neurons have been integrated into the neural circuits and are involved in memory and spatial learning.

## Figures and Tables

**Figure 3:**
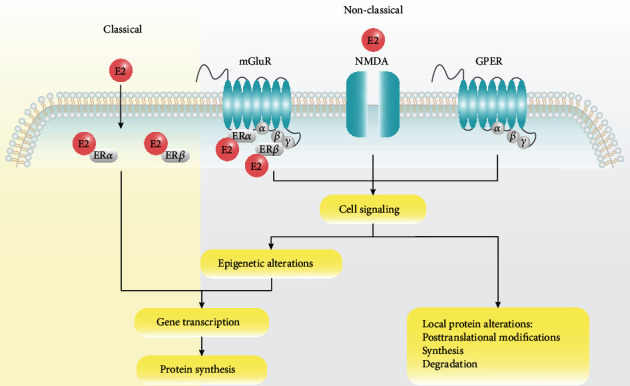
Genomic and nongenomic mechanisms underlying the regulatory effects of estrogen on neurogenesis. Classical receptors, ER*α* and ER*β*, are located in the cytoplasm or nucleus and induce genomic effects when combined with estradiol. Membrane-associated receptors, GPER, mGluR, and NMDA, are localized in the plasma membrane of neurons (e.g., tether proteins on cell membranes) and trigger rapid nongenomic effects primarily through kinase activation. Abbreviations: E2: estradiol; ER: estrogen receptor; mGluR: metabotropic glutamate receptor; NMDA: N-methyl-D-aspartate; GPER: G protein-coupled estrogen receptor.

**Table 1:** Effects of different estrogen and progesterone treatments on the adult hippocampal neurogenesis of mammals.

Treatment	Model organism	Neuron proliferation	Neuron survival	Reference
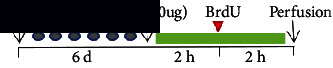	SD rats	↑		[58]
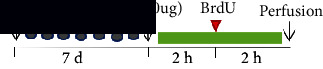		↑		
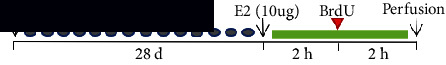	SD rats	↔		[59]
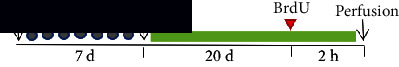		↔		
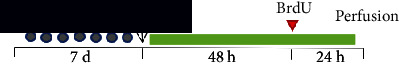	SD rats	↓		[60]
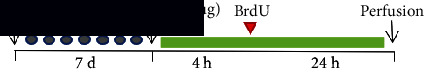	SD rats	↑		[61, 62]
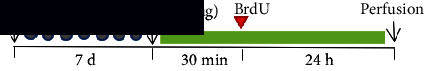	SD rats	↑		[61, 63]
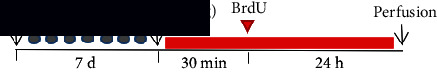		↑		
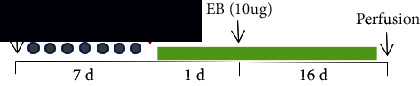	SD rats	↑	↓	[64]
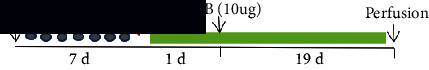	SD rats	↔	↑	[65]
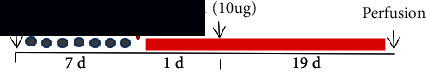		↔	↓	
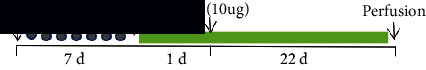	SD rats	↔	↓	[66]
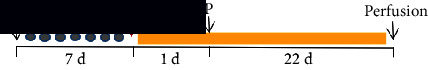		↔	↔	
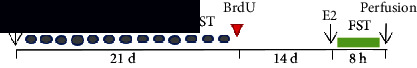	Wistar rats	↔	↔	[67]
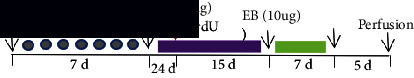	ICR mice	↓	↓	[31]
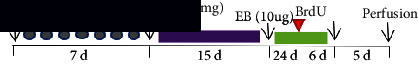		↔		
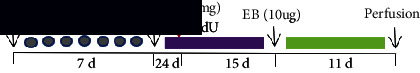		↑	↑	
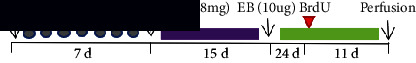		↑		

Note: “OVX” indicates bilateral ovarian resection; the green bar indicates estrogen or estradiol benzoic acid; the red bar indicates estrone; the orange bar indicates progesterone; the purple bar indicates benzoic acid and progesterone. The up arrows indicate upregulation, the down arrows indicate downregulation, and the horizontal bars indicate no change. Abbreviations: E2: estradiol; BrdU: bromodeoxyuridine; EB: estradiol benzoate; FST: forced swimming test; P4: progesterone; SD rats: Sprague-Dawley rats.

## Data Availability

All data and material files are available upon request.
